# The Effect of Physical Therapy on Regional Lung Function in Critically Ill Patients

**DOI:** 10.3389/fphys.2021.749542

**Published:** 2021-09-20

**Authors:** Christine Eimer, Katharina Freier, Norbert Weiler, Inéz Frerichs, Tobias Becher

**Affiliations:** Department of Anaesthesiology and Intensive Care Medicine, University Medical Centre Schleswig-Holstein, Kiel, Germany

**Keywords:** electrical impedance tomography, regional lung function, physical therapy, early mobilization, critical illness, alveolar recruitment, end-expiratory lung volume

## Abstract

Early mobilization has become an important aspect of treatment in intensive care medicine, especially in patients with acute pulmonary dysfunction. As its effects on regional lung physiology have not been fully explored, we conceived a prospective observational study (Registration number: DRKS00023076) investigating regional lung function during a 15-min session of early mobilization physiotherapy with a 30-min follow-up period. The study was conducted on 20 spontaneously breathing adult patients with impaired pulmonary gas exchange receiving routine physical therapy during their intensive care unit stay. Electrical impedance tomography (EIT) was applied to continuously monitor ventilation distribution and changes in lung aeration during mobilization and physical therapy. Baseline data was recorded in the supine position, the subjects were then transferred into the seated and partly standing position for physical therapy. Afterward, patients were transferred back into the initial position and followed up with EIT for 30 min. EIT data were analyzed to assess changes in dorsal fraction of ventilation (%dorsal), end-expiratory lung impedance normalized to tidal variation (ΔEELI), center of ventilation (CoV) and global inhomogeneity index (GI index).Follow-up was completed in 19 patients. During exercise, patients exhibited a significant change in ventilation distribution in favor of dorsal lung regions, which did not persist during follow-up. An identical effect was shown by CoV. ΔEELI increased significantly during follow-up. In conclusion, mobilization led to more dorsal ventilation distribution, but this effect subsided after returning to initial position. End-expiratory lung impedance increased during follow-up indicating a slow increase in end-expiratory lung volume following physical therapy.

## Summary

Physiotherapy and the concept of early mobilization has become increasingly important in the ICU. Previous studies have shown a positive effect in terms of shorter hospital stay and improved functional status at discharge. The physiological effect on the lungs has not been investigated so far, therefore, we wanted to explore the effect on lung function during a 15-min physiotherapy session with a 30-min follow-up period.

In our observational, prospective study, we included 20 spontaneously breathing patients with impaired pulmonary gas exchange during their intensive care unit stay after surgery. Using non-invasive functional lung imaging with electrical impedance tomography (EIT), we examined regional lung function of the patients before, during, and after a physiotherapy session. EIT allowed continuous assessment of the distribution of ventilation in the lungs.

We observed that ventilation of the posterior lung segments increased significantly during physiotherapy, when patients assumed a sitting and partially standing position. After the patients returned to the initial lying position before physiotherapy, the observed effect subsided immediately. Nevertheless, the follow-up showed a slow increase in end-expiratory lung volume.

## Introduction

Serious illness, long-term immobilization and mechanical ventilation are major risk factors that are associated with respiratory complications and muscular weakness (De Jonghe et al., 2007; Ali et al., 2008). Hodgson et al. (2015) showed that around 50% of mechanically ventilated patients with a ventilation duration > 48 h develop ICU- acquired weakness. For this reason, early mobilization has become a major topic in the treatment of critically ill patients. Schweickert et al. (2009) showed the positive effects of rehabilitation regarding physical and mental functions of mechanically ventilated patients. Several other studies have reported similar results in reducing the negatives effects associated with critical illness and intensive care unit (ICU) treatment (Schaller et al., 2016; Denehy et al., 2017). Early mobilization results in better functional outcomes at hospital discharge, a shorter duration of delirium and more ventilator-free days compared to standard care (Schweickert et al., 2009). Physical exercise on the intensive care unit includes sitting, standing, a range of motion exercises and passive exercises, depending on the capabilities of the individual patient (Gosselink et al., 2008).

The aims of pulmonary rehabilitation are clearing airway secretions, reducing the work of breathing, which also includes assisted cough techniques, improving respiratory function and enhancing inflation of the lungs (Gosselink et al., 2008; Jang et al., 2019). Through various exercises, atelectasis can be reduced and oxygenation and lung recruitment can be improved. Wang et al. (2018) showed that chest physiotherapy decreased extubation failure in mechanically ventilated patients in the ICU.

Despite these well-known clinical benefits, the physiological effects of early mobilization on lung function have been less well explored. The impact of early mobilization on regional ventilation, alveolar recruitment and ventilation distribution still remains unclear. Also, it has not been clarified which specific exercises, intensity, and frequency of physical therapy provide the greatest benefit. Assessing the change of lung function during physiotherapy exercise could contribute to a greater understanding of its actual benefits.

Electrical impedance tomography (EIT) is a non-invasive and radiation-free technique that enables bedside dynamic visualization of changes in regional air distribution by measuring changes in electrical impedance in cross sectional maps of the thorax (Leonhardt and Lachmann, 2012; Frerichs et al., 2017). Chest EIT thereby allows regional assessment of ventilation distribution, alveolar recruitment and atelectasis formation (Frerichs et al., 2017). The aim of the present study is to investigate the effect of physical therapy on regional lung physiology, end-expiratory lung volume and ventilation distribution with EIT.

## Methods

We conducted a prospective observational study including spontaneously breathing adult ICU patients with pulmonary impairment (Horovitz index < 300 mmHg or PaCO_2_ > 50 mmHg). In patients receiving oxygen via a nasal cannula, FiO_2_ for calculation of Horovitz index was estimated from O_2_ (l/min) as described in Supplementary Table 1. The study was carried out in three operative ICUs at a tertiary academic hospital. Exclusion criteria were patient refusal, technical limitations for EIT monitoring (chest tubes, drains, bandages, metallic implants), agitation, hemodynamic instability and BMI > 35 kg/m^2^.

The study was approved by the Ethics Committee of the Medical Faculty of the Christian-Albrechts-University Kiel, Germany (D551/20). The study was registered in the German Clinical Trials Registry (DRKS) under the reference DRKS00023076.

Primary endpoint was the change in ventilation distribution in the thoracic cross section after physiotherapy, assessed as percentage of ventilation in the dorsal lung region (%dorsal). The shift of the center of ventilation ventral-dorsal (CoV v-d) and center of ventilation right-left (CoV r-l) as well as the change of end-expiratory lung impedance (ΔEELI) were further endpoints. The secondary endpoint included the evaluation of the global inhomogeneity index (GI index) and the correlation with oxygen saturation.

Physical therapy was conducted as part of routine ICU treatment once or twice per day, beginning on the day of admission. The data described in this study were obtained during the first physiotherapy session that was performed in the morning prior to other forms of mobilization, respiratory therapy or inhalation. Before starting the physical exercise, the EIT device was applied to the rested patient. Initially, patients were motivated to perform easy warm up exercises in a recumbent bed position (30° inclination) for approximately 5 min. Depending on the capacity of the patients, movement exercises were performed with the aim of improving thoracic mobility, vital capacity, stimulation of circulation and enhancing the perception. These exercises included guided breathing, sitting up from supine, raising and lowering arms, standing up from a sitting position, and walking single steps on the spot. The intensity of exercises was dosed depending on the clinical impression of the patient, e.g., facial color, breathing rate, facial expression. After completion of the training, patients were placed in bed in the initial position. Physical therapy was performed by a trained physical therapist with an expertise in the treatment of intensive care patients.

EIT monitoring was performed using the PulmoVista 500 (Dräger Medical, Lübeck, Germany). An EIT belt with 16 integrated electrodes was placed around the patient’s thorax in the fourth to fifth intercostal space while the patient was still in supine position before mobilization. When putting on the belt, attention was paid to select the correct belt size and to individually adjust it to the width to the patient’s thorax. At least 1–3 min of undisturbed tidal breathing was recorded in this position corresponding to time point 1 (T1). Care was taken to ensure that the patient was at rest in bed at T1 and had not been mobilized previously. After transferring the patient into seated position on the edge of the bed, another EIT recording of at least 1 min duration was performed (T2). After completion of the training, but still in the seated position, EIT monitoring was continued for 2 min (T3). Time interval 4 (T4) started with the beginning of the recovery period, in which patients were back in the starting position in bed. The EIT recording was then continued for another 30 min. Based on the individual abilities of the patients, some study participants were able to mobilize to a standing position, while the others completed the exercises in a sitting position. In addition to the analyses of all patients, these two subgroups were analyzed separately.

The EIT data of time points T1–T4 were processed using the EIT Data Analysis Tool 6.1 (Dräger Medical, Lübeck, Germany) and the PulmoVista PC Software (Dräger Medical, Lübeck, Germany). Analysis was focused on the spatial distribution of ventilation. To assess regional ventilation, regions of interests (ROIs) were defined. For this purpose, the chest cross-section was divided into four equally sized quadrants (anterior right, anterior left, posterior right, posterior left). These ROIs allow to reflect regional changes in ventilation distribution by calculating ventral and dorsal shifts during T1–T4 (Pulletz et al., 2006). Dorsal percentage of ventilation (%dorsal) was calculated by summing up the values of tidal impedance variation in the posterior right and posterior let quadrants. As a more precise measure of ventilation distribution, the CoV was calculated as described by Frerichs et al. (1998). For assessment of ventilation homogeneity, the GI index was calculated. The GI index represents the degree of heterogeneity of tidal volume distribution in the lung and has proven good interpatient comparability (Zhao et al., 2009; Becher et al., 2016). CoV and GI index were evaluated during T1-T4. For assessment of GI index and CoV, Dräger EIT Data analysis tool 6.1 was used to generate a representative “minute image” for all time points, which was subsequently exported into a 32 × 32 pixel matrix. GI index and CoV were then calculated from this matrix using Microsoft Excel 2104 (Excel, Microsoft Corporation, United States). Changes in end-expiratory lung volume were assessed by analyzing the change of ΔEELI (Bodenstein et al., 2009). ΔEELI was only evaluated during 30 min of follow-up (T4) to avoid bias of the results due to the change in position.

Hemodynamic and respiratory function were continuously monitored during the intervention. Pain was evaluated before and after therapy using a numerical rating scale (NRS). In case of NRS > 4, patients received pain medication before physiotherapy. In 14 patients arterial blood gases were sampled at T1, in 7 patients at the beginning of T4 and also in 7 patients at the end of T4. In the remaining 5 patients no arterial blood gas samples were collected due to absence of an arterial line. The Horovitz index was determined in all patients. In patients without arterial line, arterial partial pressure of oxygen (PaO_2_) was derived from peripheral oxygen saturation (SpO_2_) determined by pulse oximetry to assess the Horovitz index and to evaluate gas exchange as described in Supplementary Table 2.

Statistical analysis was performed with GraphPad Prism version 9.02 (GraphPad Software, San Diego, United States) and Microsoft Excel 2104 (Excel, Microsoft Corporation, United States). The Shapiro-Wilk test for normal distribution was performed prior to the statistical analysis. Normally distributed data were described as means and standard deviation (SD) and non-normally distributed data were described as median and interquartile range. For assessment of differences in the data collected at the different time points T1–T4 and within T4, one-way ANOVA or Friedman test (according to the Gaussian distribution of data) for repeated measures was applied. Two-way ANOVA was used to compare the results of the two subgroups who were mobilized to the sitting and the standing position. When using the one- or two-way ANOVA, the Geisser-Greenhouse correction was used. Significance was set at the 0.05 probability level. To show an association between variables, Pearson correlation was used. The paired *t*-test with dependent variables was used to evaluate blood gas analyses at T1 and T4.

## Results

**FIGURE 1:**
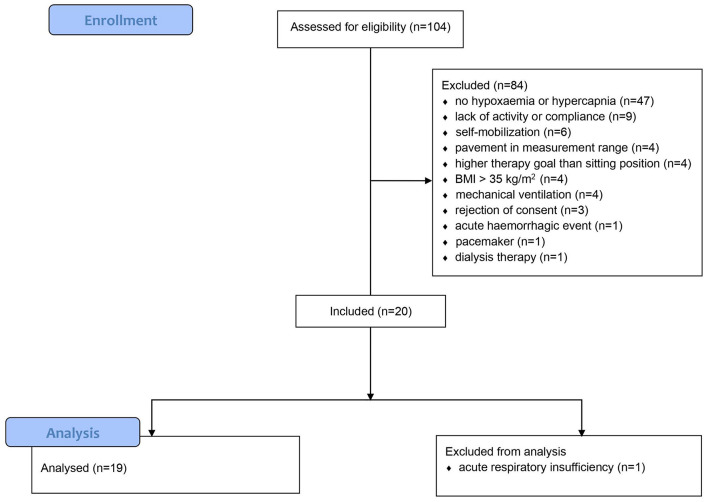
Study flow chart.

**FIGURE 2:**
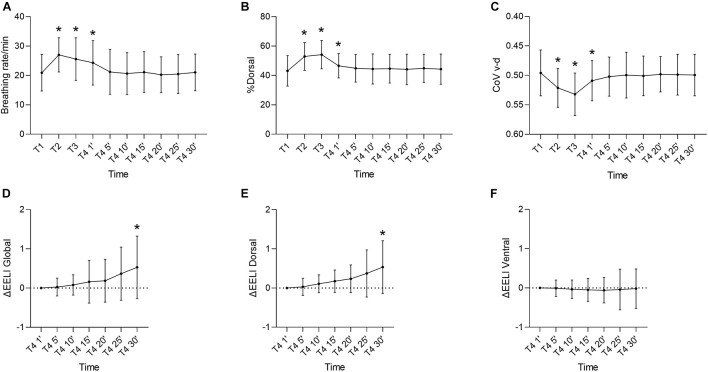
**(A)** Breathing rate. **(B)** %Dorsal. **(C)** CoV v-d. Changes from T1 (Baseline) to T2 (start sitting position), T3 (end sitting position), and T4 (follow-up total 30 min). **(D)** ΔEELI global. **(E)** ΔEELI dorsal. **(F)** ΔEELI ventral. Changes during Follow-up (T4), 1–30 min (1’–30’). Values are shown as Mean ± SD. %Dorsal, percentage of dorsal ventilation; CoV v-d, Center of ventilation ventral-dorsal; ΔEELI, change in end-expiratory lung impedance; *, presentation of significant results in relation to T1.

Out of a total of 104 screened patients, 20 patients were included in the study. In one patient, physiotherapy had to be discontinued due to acute respiratory exhaustion; this patient was excluded from the analysis (Figure 1). The demographic and clinical data are shown in Table 1. All participating patients underwent major, mostly abdominal, surgery and were admitted in the ICU for postoperative therapy and monitoring. The Horovitz index was determined in advance in all participating patients. Additionally, PaCO_2_ was determined in all patients with invasive arterial line. 9 patients had mild hypoxemia and another 9 patients suffered from moderate hypoxemia, one patient had a hypercapnia with PaCO_2_ above 50 mmHg. All patients received supplementary oxygen, 17 patients via nasal cannula, 2 patients via high-flow oxygen therapy. The average fraction of inspired oxygen (FiO_2_) was 39% (SD ± 9). One patient was tracheotomized due to cervical surgery. The median duration of mechanical ventilation before study inclusion was 8 (4–22) h. A median of 41 (18–96) h had elapsed since extubation. All participating patients had spent a median of 5 (2–12) days in the ICU prior to study inclusion. The mean respiratory rate is shown in Figure 2A.

**TABLE 1:** Demographic and clinical characteristics and Non-EIT-measurements.

Demographic and clinical characteristics	
Patients, n	19
Age, y	63 ± 13
Male	13 (68)
BMI, kg/m^2^	28 ± 5
**Medical/surgical admission, n (%)**	
Abdominal surgery	12 (64)
Thoracic surgery	1 (5)
Maxillofacial surgery	1 (5)
Vascular surgery	2 (11)
Trauma surgery	1 (5)
Spine surgery	1 (5)
Urological surgery	1 (5)
APACHE II score	8 ± 3
SOFA score	2 [2–5]
SAPS II score	29 [15–36]
Time since extubation, h	41 [18–96]
FiO_2_ (%)	39 ± 9
Horovitz index, FiO_2_/PaO_2_	229 [164–280]
ICU length of stay, Time point of EIT-measurement, d	3 [2–5]
ICU length of stay, total, d	5 [2–12]
Ventilation duration, Time point of EIT-measurement, h	8 [4–22]
Ventilation duration, total, h	9 [7–30]
**Non-EIT-measurements**	
Length of sitting (T2–T3), min	16 ± 4
SpO_2_ T1, %	96 ± 3
SpO_2_ T4 1’, %	95 ± 3
SpO_2_ T4 30’, %	96 ± 3
Breathing rate T1, breaths/min	21 ± 6
Breathing rate T4 1’, breaths/min	24 ± 8
Breathing rate T4 30’, breaths/min	21 ± 6
PaO_2_ T1 (*n* = 14), mmHg	85 ± 16
PaO_2_ T4 (*n* = 14), mmHg	84 ± 15
PaCO_2_ T1 (*n* = 14), mmHg	40 ± 6
PaCO_2_ T4 (*n* = 14), mmHg	41 ± 8

*Data are presented as n, Percent (%), Mean ± SD or Median [interquartile range].BMI, Body Mass Index; APACHE II, Acute Physiology and Chronic Health Evaluation II; SOFA, Sequential Organ Failure Assessment; SAPS I, Simplified Acute Physiology Score II; FiO*_2_, *Fraction of Inspired Oxygen; ICU, Intensive Care Unit; EIT, Electrical Impedance Tomography; SpO_2_, Peripheral Capillary Hemoglobin Oxygen Saturation; PaO_2_, Partial Pressure of Oxygen; PaCO_2_, Partial Pressure of Carbon Dioxide.*

An arterial blood gas analysis (ABG) was performed in 14 patients, 5 patients did not have an invasive blood pressure measurement established. The first ABG was taken at a median of 17 (5–24) min before T1. The second ABG was taken at a median of 11 (0–30) min during follow-up (T4). PaO_2_ in the first ABG was 85 (SD ± 16) mmHg, and the second was 84 (SD ± 15) mmHg, a difference that was not statistically significant (*p* = 0.93). PaCO_2_ was 40 (SD ± 6) mmHg in the first ABG and 41 (SD ± 8) mmHg in the second, which was also not statistically significant (*p* = 0.74).

Mobilization was performed as described in “Methods” section. All patients were mobilized to the sitting position, with 12 of 19 patients additionally mobilized to the standing position. The training time in the sitting position was 16 min in average (SD ± 4 min). Patients were then mobilized back to bed and resumed the starting supine position.

ROI-based evaluation of lung sections showed a significant increase in %dorsal, especially after transfer to the sitting position (T1–T2). The increased dorsal ventilation also continued to increase with the length of sitting (T2–T3). After transfer from the sitting position to the supine position, %dorsal started to decrease again in favor of a more ventral ventilation distribution (T3-T4 1’). Yet, the %dorsal was still significantly increased in the first minute of follow-up (T4 1’) compared to baseline (T1). From T4 5’, %dorsal showed no significant difference from baseline (T1) and remained constant (T4 5’–T4 30’) (Figure 2B).

The values of CoV ventral-dorsal showed a behavior similar to %dorsal. There was significant dorsal shift of CoV after patient was transferred into the vertical position (T1–T2). This shift increased further during the upright position (T2–T3). After the patient returned to the supine position, this effect persisted until the first minute of follow-up (T3–T4 1’). From the fifth minute of follow-up, the values approached the initial value of baseline T1 and showed no significant difference (T4 5’–T4 30’) (Figure 2C).

The result of CoV right-left measurement showed no significant differences. The mean CoV right-left was minimally shifted to the left side (*p* = 0.47). There was a positive trend after the patient was transferred to the sitting position toward the center, however without significance. The GI index showed no significant changes during the study period (*p* = 0.36).

ΔEELI was measured only during follow-up (T4 1’–T4 30’). During this time period, a significant increase of ΔEELI global was observed. Comparing the distribution of ΔEELI global in ΔEELI dorsal also showed an increase. Remarkably, seven of 19 patients showed an increase of more than 0.5, while the remaining patients showed a smaller increase. Correlations regarding Horovitz index, BMI, age, ventilation time or APACHE II score did not show a significant result. In contrast to ΔEELI dorsal, ΔEELI ventral did not change significantly during follow-up (*p* = 0.79) (Figures 2D–F).

**FIGURE 3:**
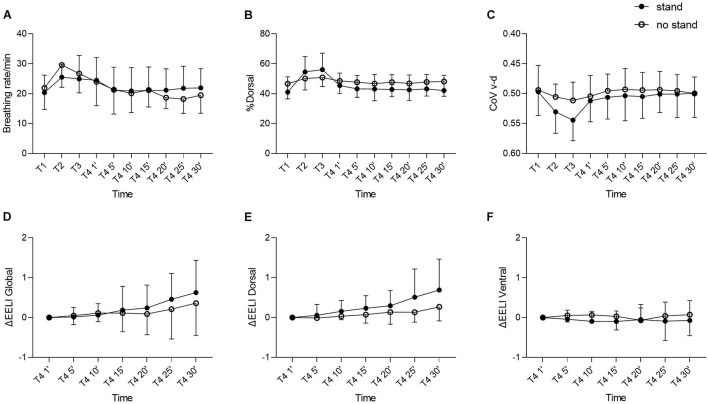
**(A)** Breathing Rate. **(B)** %Dorsal. **(C)** CoV v-d. **(D)** ΔEELI global. **(E)** ΔEELI dorsal. **(F)** ΔEELI ventral. Comparison of the results of the standing and no standing subgroups during the 30-min follow-up after the mobilization. All panels show mean values with SD. %Dorsal, percentage of dorsal ventilation; CoV v-d, center of ventilation ventral-dorsal; ΔEELI, change in end-expiratory lung impedance.

For a more comprehensive analysis, the subjects and their results were divided into two subgroups, the subjects who were mobilized into the stand and those who were not. Comparison of subgroup results between stand and no stand showed no significant difference [%dorsal (*p* = 0.5), CoV v-d (*p* = 0.44), breathing rate (*p* = 0.94), ΔEELI global (*p* = 0.64), ΔEELI dorsal (*p* = 0.18), ΔEELI ventral (*p* = 0.49)]. Descriptive results at time points T2 and T3 exhibited a greater increase in subjects who were mobilized to the stand than those who were not (Figures 3A–F).

## Discussion

The primary result of this prospective observational study is that sitting position and physiotherapy led to increased ventilation of the dorsal lung areas while applied. The increase in dorsal ventilation subsided quickly after the end of physical therapy and translated into a slow (and presumably longer lasting) increase in dorsal lung aeration.

Only spontaneously breathing patients were included in this study. Patients with severe respiratory disorder were not included because none of these patients met the inclusion criteria as they were usually mechanically ventilated. As the resilience of mechanically ventilated patients is usually lower, early mobilization for these patients consists of lighter and shorter exercises and is therefore not necessarily comparable to the training we chose for our study. Hickmann et al. (2021) showed that an upright position and early mobilization improved ventilation distribution and gas exchange especially in severely hypoxemic subjects, some of whom were also mechanically ventilated. Thus, it remains unclear whether the effects we observed in our study might have been different in patients with severely impaired gas exchange.

We must point out that the PaO_2_ estimates that were derived from SaO_2_ measurements may have led to some inaccuracies in approximating the Horovitz quotient of included patients. This approximate value was used for assessing the inclusion criterion “PaO_2_/FiO_2_ < 300 mmHg.” Therefore, we cannot rule out the possibility that some included patients may have had Horovitz quotients above this threshold.

Early mobilization is standard care for ICU patients and the selection of exercises is based on the patient′s individual abilities (Schweickert et al., 2009; Morris et al., 2016; Schaller et al., 2016). ICU mobility programs or concepts such as enhanced recovery after surgery and fast track are increasingly implemented and show a reduction in hospital length of stay and the rate of complications (Ni et al., 2013; Souza Possa et al., 2014; Fraser et al., 2015; Morgan et al., 2016; Schujmann et al., 2020). In our study, we observed real-time improvement in regional ventilation during mobilization, especially in dorsal sections of the lung. Other studies support the finding that an upright body position has physiologically beneficial effects on lung function: Adopting a semi-recumbent or sitting position of the patient at the ICU shows an improvement of lung volume and benefits in terms of gas exchange (Richard et al., 2006; Dellamonica et al., 2013). In a trial with healthy elderly people, it was shown that the PaO_2_ in the sitting position was consistently higher than in the supine position (Hardie et al., 2002). The sitting position also reduced respiratory drive compared with the supine position which might also be supportive in breathing trials (Chang et al., 2004; Walterspacher et al., 2018). In particular, Chang et al. (2004) revealed that vertical positioning with a tilt table increased minute ventilation, tidal volume and gas exchange during intervention, but there were no improvements in gas exchange post-tilt. Several studies have shown the straighter and more upright the posture the higher the forced vital capacity and forced expiratory volume in 1 s will increase (Meysman and Vincken, 1998; Stewart et al., 2000; Vilke et al., 2000; Ceridon et al., 2011).

Similar exercises were performed within our study, but the training was not always completely identical, so ultimately 19/19 patients could be mobilized to sitting but only 12/19 patients could be mobilized to standing. The comparison of the results between the patients who stood and those who did not stand during physiotherapy showed a non-significant trend toward more dorsal ventilation and toward a greater increase in dorsal end-expiratory lung volume in the patients mobilized to standing. Zafiropoulos et al. (2004) observed an improvement of the tidal volume, frequency of respiration and minute volume after changing from supine to upright posture, but no further significant effects were detected when subjects walked on the spot for 1 min. Schujmann et al. (2020) showed that patients who participated in an ICU mobility program, whose protocol included mobilization to standing position, had a better functional outcome at discharge than the control group. Hickmann et al. (2021) showed that sitting in combination with active exercise resulted in better lung aeration. The question arises whether mobilization into standing brings a significant additional gain during physiotherapy than just sitting. This specific question has not yet been captured in the current state of studies and further research is needed. It cannot be exactly clarified to what extent different exercises and their intensity contribute to lung function in our study population. Moreover, we must emphasize that mobilization of patients to a standing position may have additional beneficial effects that cannot be assessed with EIT. These include circulatory training and activation of physical perception.

ABG were analyzed before and after physical therapy, showing no difference in oxygenation and decarboxylation. Based on previous studies showing an improvement of oxygenation after mobilization (Manzano et al., 2008), we had expected to see an improvement in gas exchange within the context of alveolar recruitment. However, the time points of ABG collected in the studied patients were heterogeneous, which makes the results difficult to compare.

Body movements during physical therapy may introduce artifacts and potentially affect EIT measurements. Vogt et al. (2016) visualized the impact of movement of torso and arms on end-expiratory impedance and documented a significant influence on the measurement of EIT. Therefore, strict adherence to the upright position during measurement is recommended. For our evaluation only measurement results obtained during a resting phase were used. Care was taken to ensure that patients did not move during these phases. It cannot be evaluated whether wearing the EIT belt may have led to reduced ventilation caused by a minor decrease in thoracic compliance.

The results of %dorsal and CoV v-d imply that from the beginning of mobilization, dorsal regions of the lung were increasingly involved in ventilation. This effect was even more pronounced at the end of mobilization (T3) as compared to beginning of mobilization (T2). Interestingly, it subsided after the fifth minute of follow-up in the initial position (usually supine). Simultaneously, we found an increase in ΔEELI global and dorsal during follow-up, with ΔEELI ventral remaining unchanged. Since ΔEELI can be interpreted as an increase in end-expiratory lung volume, an increase especially in dorsal end-expiratory lung volume during follow-up was detected—in contrast to the decrease in ROI %dorsal and CoV v-d (Hinz et al., 2003). It remains unclear whether the increase is an actual gain in end-expiratory lung volume due to the upright position and physiological exercises or whether it is only a compensation of the lost lung volume due to increased activity during T2 and T3. In practical terms, the indication for more frequent mobilization or longer sitting intervals could be derived from these findings, although further studies are needed to evaluate the greatest possible benefit.

### Limitations

Our study has several limitations. The design was purely observational, so due to lack of randomization, the causality between the observed effects and physical therapy can only be assumed but cannot be proven. The sample size was small, and the patients were very heterogeneous, especially regarding the Horovitz index, surgical reason for admission and previous ventilation duration. In this respect, an increase in the number of cases could be useful in follow-up studies. In our observational study, ABG samples were taken as per routine clinical practice, which was not necessarily precisely linked to the time points T1–T4. In a future randomized study, ABG sampling should optimally be linked to T1–T4. Regarding the increase of EELI, a prognostic correlation would have been desirable. As another limitation, the study design did not allow us to differentiate whether the observed effects were provided by physiotherapy or solely by the change of position.

## Conclusion

In critically ill patients without mechanical ventilation, physical therapy in the sitting or standing position leads to an immediate redistribution of ventilation to the dorsal lung areas during therapy, which subsides quickly at the end of therapy and is followed by a prolonged and predominantly dorsal increase in end-expiratory lung aeration.

## Data Availability Statement

The raw data supporting the conclusions of this article will be made available by the authors in fully anonymized form upon reasonable request.

## Ethics Statement

The studies involving human participants were reviewed and approved by the Ethik-Kommission, Christian-Albrechts-University, Medical Faculty, University Medical Center Schleswig-Holstein, Campus Kiel, Germany. Written informed consent for participation was not required for this study in accordance with the national legislation and the institutional requirements.

## Author Contributions

CE, IF, TB, and NW: conceptualization, methodology and project administration, supervision, and validation. KF, CE, and NW: data curation. KF, CE, IF, TB, and NW: formal analysis, resources, writing—review and editing. KF and CE: writing—original draft preparation. All authors critically reviewed and approved the final submitted version of the manuscript and agreed both to be personally accountable for the author’s own contributions and to ensure that questions related to the accuracy or integrity of any part of the work, even ones in which the author was not personally involved, are appropriately investigated, resolved, and the resolution documented in the literature.

## Conflict of Interest

All authors have provided information on potential conflicts of interests directly or indirectly related to the work submitted in the journal’s disclosure forms. IF has received funding from the European Commission (Projects CRADL, under grant 668259, and WELMO, under grant 825572) and speaking and congress fees from Drägerwerk AG & Co., KGaA. TB has received funding from the European Commission (Project CRADL, under grant 668259) and lecture fees from Drägerwerk GmbH & Co., KGaA, Sedana Medical and Löwenstein Medical AG.

## Publisher’s Note

All claims expressed in this article are solely those of the authors and do not necessarily represent those of their affiliated organizations, or those of the publisher, the editors and the reviewers. Any product that may be evaluated in this article, or claim that may be made by its manufacturer, is not guaranteed or endorsed by the publisher.
