# Supplementary material for: The Effect of Physical Therapy on Regional Lung Function in Critically Ill Patients
Source: Front Physiol. 2021 Sep 20;12:749542. doi: 10.3389/fphys.2021.749542 (PMC8488288; doi:10.3389/fphys.2021.749542)
Supplement: Supplementary file 1 [file Data_Sheet_1.pdf]

The effect of physical therapy on regional lung function in critically ill patients

A prospective observational study

Christine Eimer[1†] (christine.eimer@uksh.de), **Katharina Freier**[1†] (katharinafreier@gmx.de) , Norbert Weiler[1] (norbert.weiler@uksh.de), Inéz Frerichs[1] (inez.frerichs@uksh.de), Tobias Becher[1] (tobias.becher@uksh.de)

Supplementary material

(1)

O_2 (l/min) for nasal cannulae	Estimated FiO_2 (%)
1	24
2	28
3	32
4	36
5	40
6	44

Supplementary table S1. This table was used for estimating inspired fraction of oxygen (FiO_2) in patients receiving oxygen therapy via nasal cannula.

(2)

SpO_2 (%)	Estimated PaO_2 (mmHg)
80	44
81	45
82	46
83	47
84	49
85	50
86	52
87	53
88	55
89	57
90	60
91	62
92	65
93	69
94	73
95	79
96	86
97	96
>98	>100

Supplementary table S2. This table was used for converting measured values for peripheral oxygen saturation (SpO_2) into estimated values of arterial partial pressure of oxygen (PaO_2) in patients without an arterial line. The estimated PaO_2 values were used for evaluating the inclusion criterion "Horovitz quotient below 300 mmHg".

https://www.uni-regensburg.de/medizin/dacapo-ards/medien/konversionstabelle.pdf
